# Supplementary material for: Comparative Transcriptome-Based Mining of Genes Involved in the Export of Polyether Antibiotics for Titer Improvement
Source: Antibiotics (Basel). 2022 Apr 29;11(5):600. doi: 10.3390/antibiotics11050600 (PMC9138065; doi:10.3390/antibiotics11050600)
Supplement: Supplementary file 1 [file antibiotics-11-00600-s001.zip › antibiotics-1676611-supplementary.pdf]

Supplementary Material

Comparative transcriptome-based mining of genes involved in the export of polyether antibiotics for titer improvement

Xian Liu[1,2], Yuanting Wu[1,2], Xiaojie Zhang[1,2], Qianjin Kang[1,2], Yu-Si Yan[1,3,*], Linquan Bai[1,2,*]

[1] State Key Laboratory of Microbial Metabolism, Shanghai-Islamabad-Belgrade Joint Innovation Center on Antibacterial Resistances, School of Life Sciences & Biotechnology, Shanghai Jiao Tong University, Shanghai 200240, China

[2] Joint International Research Laboratory of Metabolic & Developmental Sciences, Shanghai Jiao Tong University, Shanghai 200240, China.

[3] Institute of Biopharmaceuticals, Taizhou University, Taizhou 318000, China

Table S1. Strains and plasmids used in this study.

Strains or plasmids	Features	Sources
Streptomyces albus		
BK3-25	Salinomycin high-yield producer	[1]
LX01	BK3-25Δ*SLNHY_0929*	This study
LX02	BK3-25Δ*SLNHY_1893*	This study
LX03	BK3-25Δ*SLNHY_3363*	This study
LX04	BK3-25Δ*SLNHY_4037*	This study
LX05	BK3-25Δ*SLNHY_0199*	This study
LX06	BK3-25Δ*SLNHY_0818*	This study
LX07	BK3-25Δ*SLNHY_6316*	This study
LX08	BK3-25Δ*SLNHY_6652*	This study
LX09	BK3-25::*SLNHY_0929*	This study
LX-10	BK3-25::*SLNHY_1893*	This study
LX-11	BK3-25::*SLNHY_3363*	This study
LX-12	BK3-25::*SLNHY_4037*	This study
LX-13	BK3-25::*SLNHY_0199*	This study
LX-14	BK3-25::*SLNHY_0818*	This study
LX-15	BK3-25::*SLNHY_6316*	This study
LX-16	BK3-25::*SLNHY_6652*	This study
LX-17	LX01::*SLNHY_0929*	This study
LX-18	LX02::*SLNHY_1893*	This study

LX-19	LX03::*SLNHY_3363*	This study
LX-20	LX04::*SLNHY_4037*	This study
LX-21	LX05::*SLNHY_0199*	This study
LX-22	LX06::*SLNHY_0818*	This study
LX-23	LX07::*SLNHY_6316*	This study
LX-24	LX08::*SLNHY_6652*	This study
LX-25	BK3-25::pIB139	This study
Streptomyces lividans		
TK24		[2]
LX-26	TK24::pIB139	This study
LX-27	TK24::*LNHY_0929*	This study
LX-28	TK24::*SLNHY_1893*	This study
LX-29	TK24::*SLNHY_3363*	This study
LX-30	TK24::*SLNHY_4037*	This study
LX-31	TK24::*SLNHY_0199*	This study
LX-32	TK24::*SLNHY_0818*	This study
LX-33	TK24::*SLNHY_6316*	This study
LX-34	TK24::*SLNHY_6652*	This study
Streptomyces cinnamonensis		
ATCC 15413		[3]

LX-35	ATCC 15413::pLQ648	This study
LX-36	ATCC 15413::*SLNHY_0929*	This study
LX-37	ATCC 15413::*SLNHY_1893*	This study
LX-38	ATCC 15413::*SLNHY_3363*	This study
LX-39	ATCC 15413::*SLNHY_4037*	This study
Streptomyces lasaliensis		
ATCC 31180		[4]
LX-40	ATCC 31180::pLQ648	This study
LX-41	ATCC 31180::*SLNHY_0929*	This study
LX-42	ATCC 31180::*SLNHY_1893*	This study
LX-43	ATCC 31180::*SLNHY_3363*	This study
LX-44	ATCC 31180::*SLNHY_4037*	This study
Streptomyces hygroscopicus		
XM201-*ga32*	XM201 with geldanamycin BGC deleted	[5]
LX-45	XM201-*ga32*::pLQ648	This study
LX-46	XM201-*ga32*::*SLNHY_0929*	This study
LX-47	XM201-*ga32*::*SLNHY_1893*	This study
LX-48	XM201-*ga32*::*SLNHY_3363*	This study
LX-49	XM201-*ga32*::*SLNHY_4037*	This study

E. coli		
DH10B	F′ (*traΔ36 proAB lacIq lacZΔM15*) *rpsL* (*strR*) *thr leu endA thi-1 lacY galK galT ara tonA tsx dam dcm supE44 Δ*(*lac-proAB*) *Δ*(*mcrCmrr*)102::Tn10 (*tetR*)	GIBCO-BRL
ET12567(pUZ8002)	*RecE dam dcm hsds Cm Str Tet Km*	[6]
**Plasmids**		
pBluescript II SK+	*bla lacZ orif1*	Strategene
pIB139	*ΦC3I int aac(3)IV lacZa* $oriT_{RK2}$ *ermE**p	[7]
pLQ646	*ΦC3I int aac(3)IV lacZa* $oriT_{RK2}$ *kasO*p*	[8]
pJTU1278	*pIJ101-rep tsr oriT*	[9]
pLQ1250	Insertion of *SLNHY_0929* downstream of *ermE**p in pIB139	This study
pLQ1251	Insertion of *SLNHY_1893* downstream of *ermE**p in pIB139	This study
pLQ1252	Insertion of *SLNHY_3363* downstream of *ermE**p in pIB139	This study
pLQ1253	Insertion of *SLNHY_4037* downstream of *ermE**p in pIB139	This study
pLQ1254	Insertion of *SLNHY_0199* downstream of *ermE**p in pIB139	This study

pLQ1255	Insertion of *SLNHY_0818* downstream of *ermE**p in pIB139	This study
pLQ1256	Insertion of *SLNHY_6316* downstream of *ermE**p in pIB139	This study
pLQ1257	Insertion of *SLNHY_6652* downstream of *ermE**p in pIB139	This study
pLQ1258	Insertion of *SLNHY_0929* downstream of *kasO*p* in pLQ646	This study
pLQ1260	Insertion of *SLNHY_3363* downstream of *kasO*p* in pLQ646	This study
pLQ1261	Insertion of *SLNHY_4037* downstream of *kasO*p* in pLQ646	This study
pLQ1266	Insertion of 1233 bp left arm and 1184 bp right arm of *SLNHY_0929* in pJTU1278	This study
pLQ1267	Insertion of 1191 bp left arm and 1000 bp right arm of *SLNHY_1893* in pJTU1278	This study
pLQ1268	Insertion of 1138 bp left arm and 1070 bp right arm of *SLNHY_3363* in pJTU1278	This study
pLQ1269	Insertion of 1155 bp left arm and 1068 bp right arm of *SLNHY_4037* in pJTU1278	This study
pLQ1270	Insertion of 1252 bp left arm and 1253 bp right arm of *SLNHY_0199* in pJTU1278	This study

pLQ1271	Insertion of 1248 bp left arm and 1211 bp right arm of *SLNHY_0818* in pJTU1278	This study
pLQ1272	Insertion of 1109 bp left arm and 1131 bp right arm of *SLNHY_6316* in pJTU1278	This study
pLQ1273	Insertion of 1172 bp left arm and 1091 bp right arm of *SLNHY_6652* in pJTU1278	This study

Table S2. Primers used in this study.

Primer	Sequence (5′-3′)
SLNHY_0929-LF-*Xba*I	TATATCTAGAGTAGGCGGCGTTGTTGAAG
SLNHY_0929-LR-*Hin*dIII	TATAAAGCTTTGAACAGCGTGCGGAAGT
SLNHY_0929-RF-*Hin*dIII	TATAAAGCTTCCGAACAACAGGAGGAGGTC
SLNHY_0929-RR-*Kpn*I	TATAGGTACCTGGTGAGCGAGCCGAAGTA
SLNHY_0929-YZ-F	CAGAGCAATGTGAAGGTGTGA
SLNHY_0929-YZ-R	TCCTGTGGAGTCTTGAACGA
SLNHY_1893-LF-*Xba*I	TATATCTAGACGAGGAAGAGGCGAAGGAT
SLNHY_1893-LR-*Hin*dIII	TATAAAGCTTACAGCAGATGGTGGTAGGC
SLNHY_1893-RF-*Hin*dIII	TATAAAGCTTTCGTTGGAAGAGGCGTTCA
SLNHY_1893-RR-*Kpn*I	TATAGGTACCCACCCACAGGCACATGATC
SLNHY_1893-YZ-F	ACTACGACCGCCTCAAGGA
SLNHY_1893-YZ-R	TCGCCGTACTCGTGGTTGA
SLNHY_4037-LF-*Xba*I	TATATCTAGATCGCCAAGAACATCGTCCTC
SLNHY_4037-LR-*Eco*RI	TATAGAATTCCGCTCATCGCCTTCGTCAT
SLNHY_4037-RF-*Eco*RI	TATAGAATTCAGGACGAAGGTGACCCAGA
SLNHY_4037-RR-*Hin*dIII	TATAAAGCTTCGAAGAACGACGGCAACTT
SLNHY_4037-YZ-F	GATCTCGCTCAGCTCGTTCT
SLNHY_4037-YZ-R	CCTCGTGCTCTTCGTCATCA
SLNHY_3363-LF-*Xba*I	TATATCTAGAGACGATATTGGAGACCATGCC
SLNHY_3363-LR-*Eco*RI	TATAGAATTCTGCTGACCGCCATCAAGTA

SLNHY_3363-RF-*Eco*RI	TATAGAATTCCGCTCCTTGAACCAGTCCA
SLNHY_3363-RR-*Hin*dIII	TATAAAGCTTTACCACGGCAACACCAACTA
SLNHY_3363-YZ-F	AGGACGAAGGAGATGTGGAAG
SLNHY_3363-YZ-R	GTCACCGTGGAGCACAAGTA
SLNHY_0199-LF-*Xba*I	TATATCTAGATTCTCCGGTGGCGAGTTGGG
SLNHY_0199-LR-*Eco*RI	TATAGAATTCCCGCCGCAAGGAGGTGTCCCTGTGA
SLNHY_0199-RF-*Eco*RI	TATAGAATTCCCCAGGAAACCGATGGCGAAAA
SLNHY_0199-RR-*Hin*dIII	TATAAAGCTTCGGGGAAGACTGGTGGTGGATT
SLNHY_0199-YZ-F	GGCCAGGTCGTCGCCCATCT
SLNHY_0199-YZ-R	GTGACCATTCCGTTTCTGTTTCC
SLNHY_0818-LF-*Xba*I	TATATCTAGAGGTGATGGCGACGATGTCCT
SLNHY_0818-LR-*Eco*RI	TATAGAATTCTCCCGCCGCACGCAGAAGA
SLNHY_0818-RF-*Eco*RI	TATAGAATTCTGCGCGGAGTTCTTGGCGAG
SLNHY_0818-RR-*Hin*dIII	TATAAAGCTTCACCGCGGAGATCGCGGTGC
SLNHY_0818-YZ-F	CTTGGCGGCGGCGATGTTGT
SLNHY_0818-YZ-R	CGCAACAACGGTGTCTTCAGC
SLNHY_6316-LF-*Xba*I	TATATCTAGAGCGACCAGTTGCTCGGCGGC
SLNHY_6316-LR-*Eco*RI	TATAGAATTCGGCTCCCCGGTCAACTCCTC
SLNHY_6316-RF-*Eco*RI	TATAGAATTCCGGACAGGAACCGGCGGAAGAA
SLNHY_6316-RR-*Hin*dIII	TATAAAGCTTACGCGGACCCGGCGAACGCA
SLNHY_6316-YZ-F	TCAACCTTGCCGCCGACCTG
SLNHY_6316-YZ-R	CATGCGTCCTGCCTCCTTGCT

SLNHY_6652-LF-*Xba*I	TATATCTAGACCCGGTTCGAGGCGCAGGGTGAT
SLNHY_6652-LR-*Eco*RI	TATAGAATTCCGAAGAACTGGAGGAACCC
SLNHY_6652-RF-*Eco*RI	TATAGAATTCGAGGTCCCATGACTCGGAGAAG
SLNHY_6652-RR-*Hin*dIII	TATAAAGCTTCGATGGCGGAAGAAGCGATGAC
SLNHY_6652-YZ-F	CGTCCGCAGCCACATGGTGT
SLNHY_6652-YZ-R	ATCGTCGCACCACCCTCCTG
SLNHY_0929-F-*Xba*I	TATATCTAGAATGCAGACCAACTCCCCCTG
SLNHY_0929-R-*Not*I	TATAGCGGCCGCTCAGGAATCGGCGGGTGCGG
SLNHY_1893-F-*Xba*I	TATATCTAGAATGATCGAGCTCGAAGGGCT
SLNHY_1893-R-*Not*I	TATAGCGGCCGCTCAGGCCCCCTTCCCGGGTC
SLNHY_4037-F-*Xba*I	TATATCTAGAATGAGCCACGCAGCCACCAC
SLNHY_4037-R-*Not*I	TATAGCGGCCGCTCAGCGACCGGACTGCGGGG
SLNHY_3363-F-*Xba*I	TATATCTAGAGTGGCCGTGACCGCCGCGCT
SLNHY_3363-R-*Not*I	TATAGCGGCCGCCTACTTGATGGCGGTCAGCA
SLNHY_0199-F-*Xba*I	TATATCTAGATCACAGGGACACCTCCTTGC
SLNHY_0199-R-*Not*I	TATAGCGGCCGCGTGGTTTTCGCCATCGGTTT
SLNHY_0818-F-*Xba*I	TATATCTAGATCAGGCGCGTCCCGCGGTCT
SLNHY_0818-R-*Not*I	TATAGCGGCCGCGCGATGAGCGACCTCGCCAA
SLNHY_6316-F-*Xba*I	TATATCTAGAGTGAGCGCGCCGACCGAGGA
SLNHY_6316-R-*Not*I	TATAGCGGCCGCTCAGCGCGGGACGGCCTTTC
SLNHY_6652-F-*Xba*I	TATATCTAGATCATGCGGGTTCCTCCAGTT
SLNHY_6652-R-*Not*I	TATAGCGGCCGCCCCCTTCCGTGCCGCCCGCG

SLNHY_0929-F-*Nde*I	TATACATATGATGCAGACCAACTCCCCCTG
SLNHY_0929-R-*Eco*RI	TATAGAATTCTCAGGAATCGGCGGGTGCGG
SLNHY_0929-over-YZ-R	TCAGGAATCGGCGGGTGCGG
SLNHY_4037-F-*Nde*I	TATACATATGATGAGCCACGCAGCCACCAC
SLNHY_4037-R-*Eco*RI	TATAGAATTCTCAGCGACCGGACTGCGGGG
SLNHY_4037-over-YZ-R	TCAGCGACCGGACTGCGGGG
SLNHY_3363-F-*Nde*I	TATACATATG GTGGCCGTGACCGCCGCGCT
SLNHY_3363-R-*Eco*RI	TATA GAATTCCTACTTGATGGCGGTCAGCA
SLNHY_3363-over-YZ-R	CTACTTGATGGCGGTCAGCA
pIB139-over-YZ-F	CGAGTGTCCGTTCGAGTGGCGG
pLQ646-over-YZ-F	TTTGACAACATGCTGTGCGG
SLNHY_0929-RT-F	GGACTCGCTCACCCTCGGACA
SLNHY_0929-RT-R	CGTTGGCCTGCACCAGGCCT
SLNHY_1893-RT-F	TATCCTGTCGGCGCCCCGG
SLNHY_1893-RT-R	CCAGCCTTGGCCCCAGCCGG
SLNHY_3363-RT-F	TGACCCAGACCAGCCGGTC
SLNHY_3363-RT-R	GGCGATCTTCTTCGAGGAGTT
SLNHY_4037-RT-F	CCAGCATGACGCCCGTGGTG
SLNHY_4037-RT-R	AGATGCTCAAGCTCGTCCCG
SLNHY_0199-RT-F	GCAGATCAGGCCCGGCCAGC
SLNHY_0199-RT-R	CGACTATCTGCGGTTCTGGATGG
SLNHY_0818-RT-F	TAGCCGACGGCCTTGTCGGC

SLNHY_0818-RT-R	AGTTCGTCCGCAAGATCCGC
SLNHY_6316-RT-F	CCTGGACGGCGTGAAGTTGGAC
SLNHY_6316-RT-R	GCCCAGCTCGTGGCTGATCC
SLNHY_6652-RT-F	CGGAGGCCAGCTCGGTGGCG
SLNHY_6652-RT-R	GGGTGCCTGCTCTTCCTCTTCG
hrdB-RT-F	TGGTCGAGGTCATCAACA
hrdB-RT-R	GTCACCGAACTCACTGTC

Table S3. Transcription data and annotations of eight candidate genes.

Gene	Soybean oil supplementation			Annotation
	**5%**	**10%**	**15%**	
SLNHY_0929	8.798	9.275	9.297	major facilitator transporter
SLNHY_1893	9.026	9.27	9.181	nodulation ABC transporter NodI
SLNHY_3363	8.851	12.195	11.203	ABC Fe^{3+} transporter binding protein
SLNHY_4037	10.539	10.715	10.833	phosphate ABC transporter permease
SLNHY_0199	7.499	10.286	9.437	iron ABC transporter
SLNHY_0818	9.5	9.499	9.838	ABC transporter permease protein
SLNHY_6316	9.993	10.285	10.293	ABC transporter-like protein
SLNHY_6652	9.756	9.946	10.056	ABC transporter transmembrane subunit

Table S4. RT-qPCR results of candidate genes.

**Gene**	***SLNHY_0929***	***SLNHY_1893***	***SLNHY_3363***	***SLNHY_4037***	***SLNHY_0199***	***SLNHY_0818***	***SLNHY_6316***	***SLNHY_6652***
Ct (5%)	27.63	26.65	30.38	25.36	30.66	26.43	28.51	30.22
Ct (15%)	24.56	24.23	27.52	23.99	29.42	25.52	26.68	28.45
$-\Delta Ct$ [a]	3.06	2.42	2.87	1.37	1.24	0.90	1.83	1.78
$2^{-\Delta Ct}$	8.34	5.35	7.31	2.58	2.36	1.87	3.56	3.43

[a] $-\Delta Ct$ stands for the difference value between Cts under 5% and 15% soybean oil supplementation.

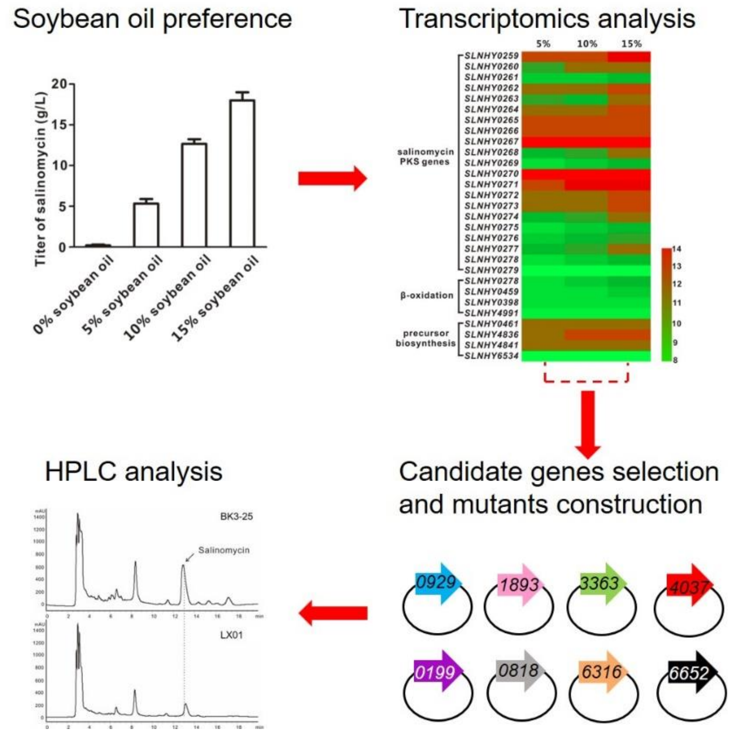

Figure S2. Flow chart of the strategy for exporter genes identification.

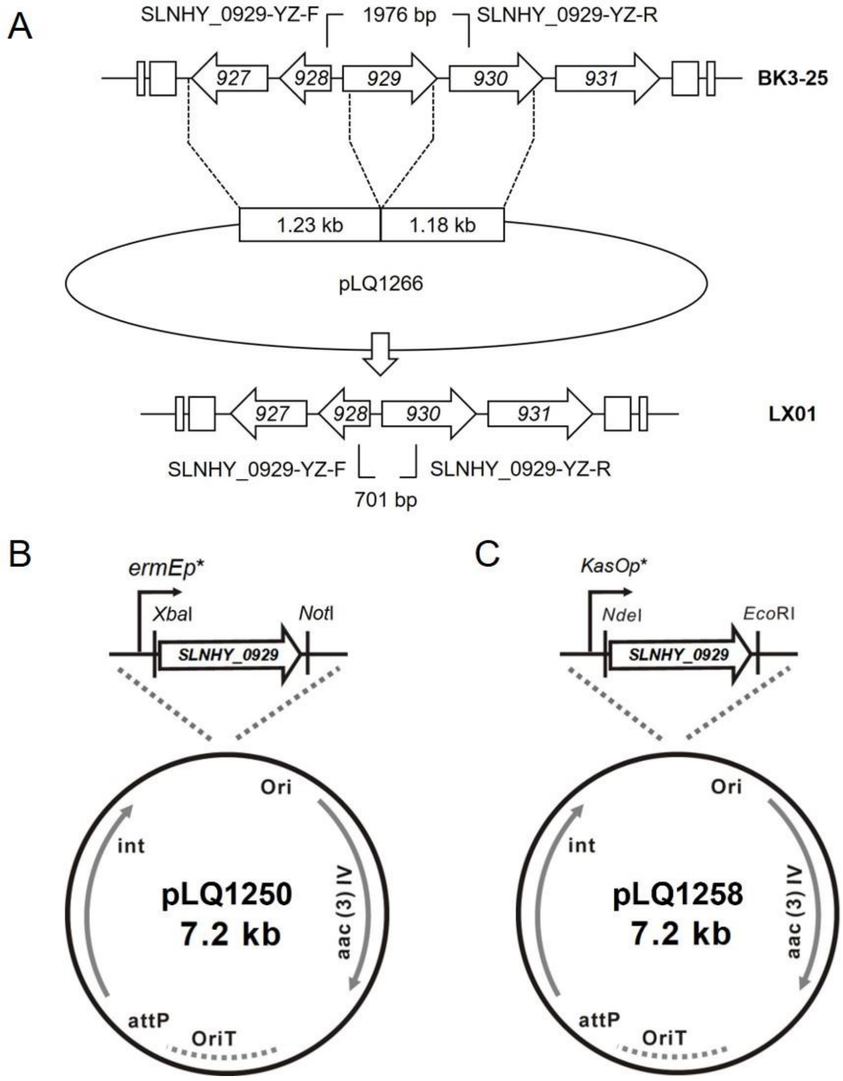

Figure S2. The process of gene deletion and over-expression of *SLNHY_0929*. (A) pLQ1266 containing upstream and downstream of *SLNHY_0929* was obtained. Through homologous recombination the gene was deleted, and the primers SLNHY_0929-YZ-F/R were used to verify the double crossover mutant LX01. (B) pLQ1250, derived from pIJ139, was obtained by inserting *SLNHY_0929* between *Xba*I and *Not*I digestion sites. (C) pLQ1258, derived from pLQ646, was obtained by inserting *SLNHY_0929* between *Nde*I and *Eco*RI digestion sites.

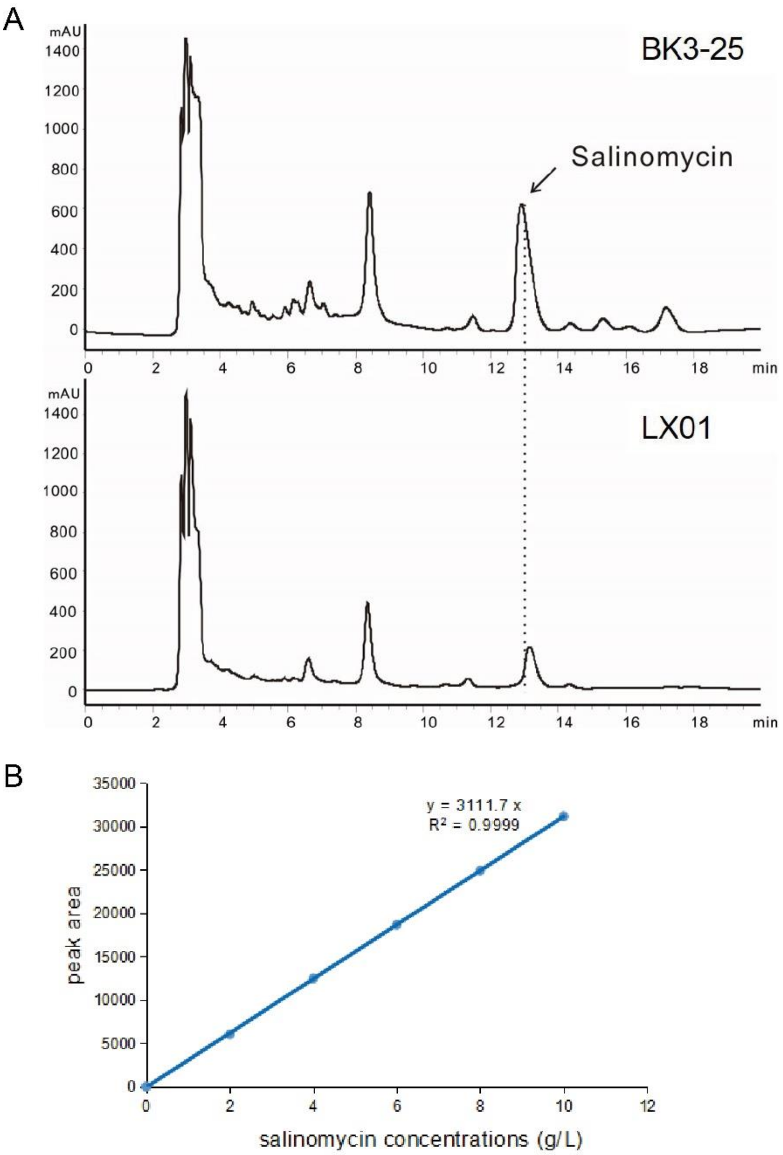

Figure S3. HPLC analysis of salinomycin in the fermentation culture of BK3-25 and its mutant LX01 with *SLNHY_0929* deleted. (A) HPLC chromatograms of 10-fold diluted fermentation culture of BK3-25 and its mutant LX01 with *SLNHY_0929* deleted. (B) The calibration curve of different concentrations of salinomycin standards. The detection UV wavelength is 210 nm.

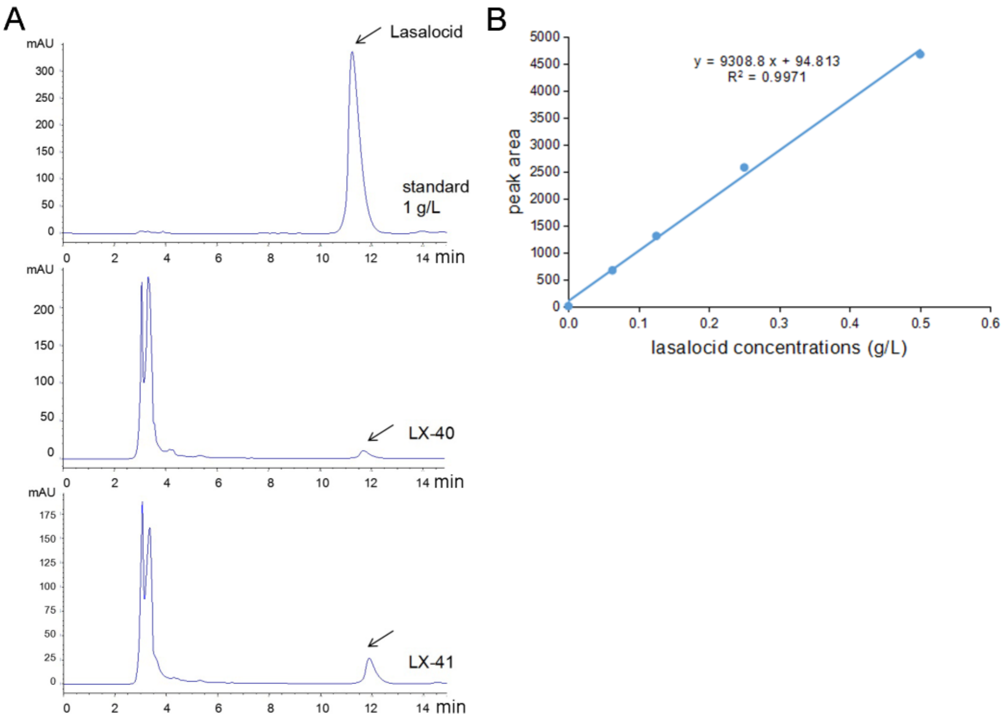

Figure S4. HPLC analysis of lasalocid in the fermentation culture of *S. lasaliensis* and its mutants. (A) HPLC analysis of lasalocid standard (1 g/L) and the 10-fold diluted fermentation culture of *S. lasaliensis* and its mutants. The detection UV wavelength is 305 nm. (B) The calibration curve of different concentrations of lasalocid standards.

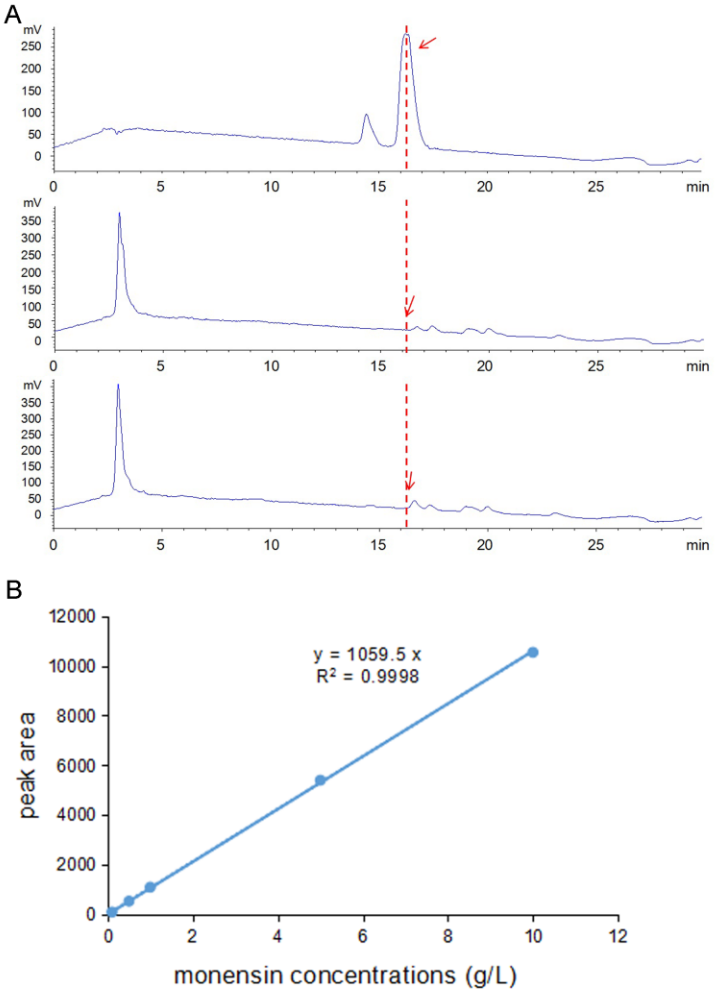

Figure S5. HPLC analysis of monensin in the fermentation culture of *S. cinnamonensis* and its mutants. (A) HPLC analysis of monensin standard (10 g/L) and the two-fold diluted fermentation culture of *S. cinnamonensis* and its mutants. (B) The calibration curve of different concentrations of monensin standards.

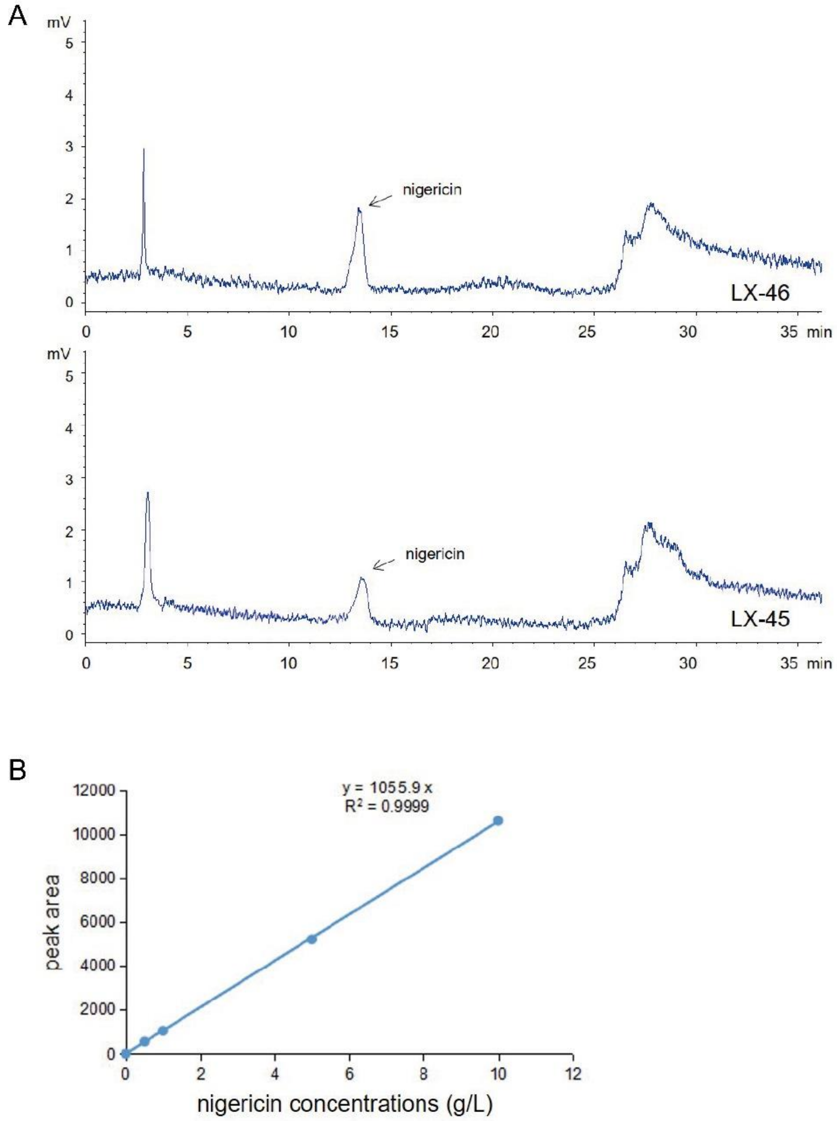

Figure S6. HPLC analysis of nigericin in the fermentation culture of *S. hygroscopicus* and its mutants. (A) HPLC analysis of two-fold diluted fermentation culture of *S. hygroscopicus* and its mutants. (B) The calibration curve of different concentrations of nigericin standards.

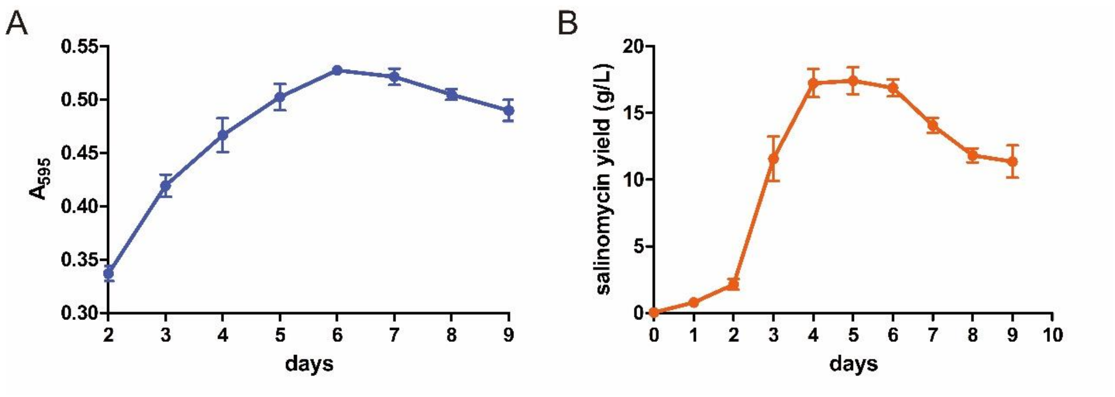

Figure S7. Growth (A) and salinomycin titer (B) curves of *Streptomyces albus* BK3-25. Due to the insoluble residues in the liquid medium, total intracellular nucleic acid was determined to represent the growth of *S. albus*. The concentration of intracellular nucleic acid was determined as described in Materials and methods. Mean values of three independent experiments with SD are indicated by error bars.

Reference

1. Zhang, X.; Lu, C.; Bai, L. Mechanism of salinomycin overproduction in *Streptomyces albus* as revealed by comparative functional genomics. *Appl. Microbiol. Biot*. **2017**, *101*, 4635–4644,.

2. Hopwood, D.A.; Hintermann, G.; Kieser, T.; Wright, H.M. Integrated DNA sequences in three streptomycetes form related autonomous plasmids after transfer to *Streptomyces lividans*. *Plasmid.* **1984**, *11*, 1–16.

3. Day, L.E.; Chamberlin, J.W.; Gordee, E.Z.; Chen, S.; Gorman, M.; Hamill, R.L.; Ness, T.; Weeks, R.E.; Stroshane, R. Biosynthesis of monensin. *Antimicrob. Agents Chemother.* **1973**, *4*, 410–414.

4. Migita, A.; Watanabe, M.; Hirose, Y.; Watanabe, K.; Tokiwano, T.; Kinashi, H.;

Oikawa, H. Identification of a gene cluster of polyether antibiotic lasalocid from *Streptomyces lasaliensis*. *Biosci. Biotechnol. Biochem.* **2009**, *73*, 169–176.

5. Wang, X.; Ning, X.; Zhao, Q.; Kang, Q.; Bai, L. Improved PKS gene expression with strong endogenous promoter resulted in geldanamycin yield increase. *J. Biotechnol.* **2017**, *12*, 1–8.

6. Smith, P. High efficiency intergeneric conjugal transfer of plasmid DNA from *Esc herichia coli* to methyl DNA-restricting streptomycetes. *FEMS. Microbiol. Lett*. **1997**, *155*, 223-229.

7. Zhang, X.; Lu, C.; Bai, L. Conversion of the high-yield salinomycin producer *Streptomyces albus* BK3-25 into a surrogate host for polyketide production. *Sci. China Life Sci*. **2017**, *60*, 1000–1009.

8. Wang, X.; Wang, R.; Kang, Q.; Bai, L. The antitumor agent ansamitocin P-3 binds to cell division protein FtsZ in *Actinosynnema pretiosum*. *Biomolecules.* **2020**, *10*, 699.

9. Wang, Z.; Bai, L.; Liang, J.; Zhou, X.; Deng, Z. Two pHZ1358 derivative vectors for efficient gene knockout in *Streptomyces*. *J. Microbiol. Biotechnol*. **2010**, *20*, 678–682.
